# Microstructural Evolution in High-Strain-Rate Deformation of Ti-5Al-5Mo-5V-1Cr-1Fe Alloy

**DOI:** 10.3390/ma11050839

**Published:** 2018-05-18

**Authors:** Chun Ran, Pengwan Chen, Zemin Sheng, Jingbo Li, Wangfeng Zhang

**Affiliations:** 1 State Key Laboratory of Explosion Science and Technology, Beijing Institute of Technology, Beijing 100081, China; 2 School of Materials Science and Engineering, Beijing Institute of Technology, Beijing 100081, China; m15090359861@163.com (Z.S.); lijb@bit.edu.cn (J.L.); 3 Beijing Institute of Aeronautical Materials, Beijing 100095, China; wfzbiam@126.com

**Keywords:** Ti-55511 alloy, forced shear tests, adiabatic shear band, dynamic recrystallization, shear band width

## Abstract

To study the microstructural evolution in high-strain-rate shear deformation of Ti-5Al-5Mo-5V-1Cr-1Fe (Ti-55511) alloy, a series of forced shear tests of hat-shaped specimens have been conducted using a split Hopkinson pressure bar combined with the “strain-frozen” technique. A localized shear band is induced in Ti-55511 alloy in these tests. The experimental results demonstrate that the flow stress in hat-shaped specimens remains constant (about 600 MPa) and is independent of punching depth. The width of the adiabatic shear band increases with increasing punching depth and tends to saturate at 30 μm, and the estimation of the adiabatic shear band (ASB) width in hat-shaped (HS) specimens has been modified. Relying on the experimental results, thermal softening has a minor effect on the onset of the adiabatic shear band and dynamic recrystallization formation, and the nucleation mechanism for dynamic recrystallization is strain-induced boundary migration and subgrain rotation and coalescence. In addition, we suggest the concept of adhesive fracture as the dynamic failure mechanism for Ti-55511 alloy.

## 1. Introduction

The term “adiabatic shear band” (hereinafter referred to as ASB) has been widely accepted by researchers since it was first mentioned in the original report of Zener and Hollomon in 1944 [1]. ASB is an important failure mechanism of solid materials in high-strain-rate deformation, especially for titanium alloys [2].

The outstanding physical properties of titanium alloys, such as high strength-to-weight ratio, good hardenability and excellent crack growth resistance, have made them into very attractive materials in aerospace, medical equipment and automotive industrial applications [3,4,5,6]. A considerable number of investigations on titanium alloys under high-strain-rate loading conditions have been conducted over the last two decades [7,8,9,10,11]. Meyers et al. [7] found a three-fold difference between measured width of ASB and predicted values, calculated by the criterion proposed by Bai et al. [12] and Dodd and Bai [13,14], in commercially pure *α*-titanium (TA2) hat-shaped (HS) specimens, while the measured width of ASB is slightly higher than the calculated results in Chen et al.’s work [15]. Nanograins with a size of 10–30 nm were observed by Rittel et al. [9], while a subgrain of approximately 200 nm was observed by Meyers et al. [7].

Ti-5Al-5Mo-5V-1Cr-1Fe (Ti-55511) alloy, a typical near-*β*-type titanium alloy, is superior as an aircraft structural material due to its 15–20% weight loss as compared to Ti-6Al-4V (TC4) alloy [16]. A considerable number of investigations on Ti-55511 alloy have been conducted over the last five years, and most of them focused on materials fabrication [17,18,19,20]. Liang et al. [21] found that the recrystallized volume fraction of Ti-55511 alloy could be quantified as the net softening effect of dynamic recrystallization (DRX) over dynamic recovery mechanisms during hot deformation. Nan et al. [22] pointed out that the influence of strain rate on DRX evolution was the major factor to determine strain-rate sensitivity. However, the findings of Liang et al. and Nan et al. are derived from low-strain-rate loading conditions (<50 s^−1^). Only a few studies have reported on dynamic mechanical properties of Ti-55511 alloy (>100 s^−1^) [16]. Although the main mechanism for adiabatic shear is the competition between the hardening effect (strain and strain rate) and thermal softening effect, the whole process is very complex and involves high strain rates, high local temperature, large plastic deformation and so forth [2]. In fact, the mechanical behavior of Ti-55511 titanium alloy is of great complexity and is strongly sensitive to the loading conditions, such as strain and strain rate [23]. The mechanical behavior and microstructural evolution of Ti-55511 alloy in the dynamic deformation process are still not well understood.

The purposes of this study are to gain deeper insights into: (a) dynamic mechanical behavior and microstructural evolution of Ti-55511 alloy in forced shear tests, and (b) the relationship of ASB width between measurement and calculation using the half-width of the shear band criterion proposed by Bai [12] and Dodd and Bai [13,14].

## 2. Materials and Methods

The Ti-55511 alloy used in the present investigation was in the form of a forged bar with a diameter of 155 mm from the Beijing Institute of Aeronautical Materials, Aero Engine Corporation of China (AECC), PR China. The *β*-transus temperature of the as-received bars is approximately 1163 K via the metallographic observation method. The chemical composition of the alloy is listed in Table 1. More information of the material has been described previously [16].

**Figure 1:**
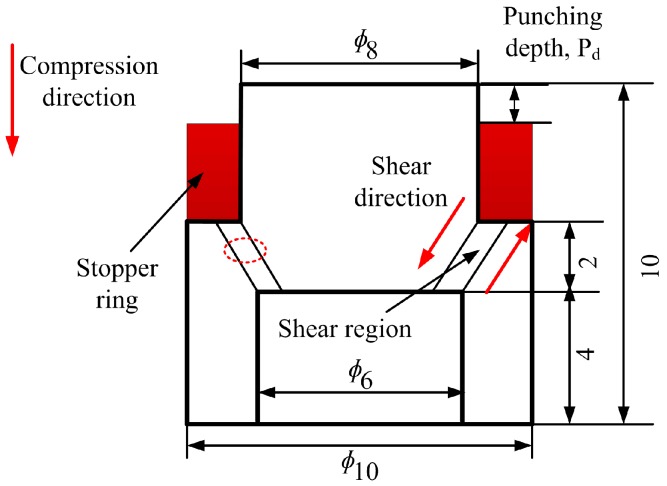
In-plane dimensions of the HS specimen (dimensions in mm).

A series of dynamic forced shear tests were carried out at 293 K by means of the split Hopkinson pressure bar (SHPB, School of Aeronautics, Northwestern Polytechnical University, Xi’an, China) technique using hat-shaped (HS) specimens [7,24]. This specially designed specimen configuration allows the creation of a well-controlled localized shear band region during deformation, and has been successfully used in the studies of large strain, high-strain-rate deformation of metals in conditions of forced localized shear [15,25,26,27]. The samples were sandwiched between the incident and transmission bars during SHPB tests. High-strength steel stopper rings were used to ensure a prescribed displacement in the principal plastic deformation region (see Figure 1). The HS specimens were machined from the forged bar using electrical discharge machining (EDM) (Jiangsu Taizhou Chuang yuan Machine Tool Co., Ltd., Taizhou, China) and a numerical control milling machine (Hebei Huayue Machinery Manufacturing Co., Ltd., Xingtai, China), where details of the experimental setup were described previously [16]. Different shear strains were obtained by varying the punching depths (*P_d_*), that is, the thickness of the stopper ring, as depicted in Figure 1. The values of *P_d_* were prescribed as 0.7, 0.8, 0.9, 1.0 and 1.1 mm, respectively. It should be pointed out that the bar–specimen interfaces were sufficiently lubricated in order to reduce friction and specimen barreling. It also should be pointed out that we just focus on the microstructural evolution of Ti-55511 alloy after ASB formed in this work, and three HS specimens were used for each loading condition. The velocity of the striker, v, can be calculated as the distance of the photo diode (a) divided by the time (t) recorded by a data acquisition instrument. The stress state in the plastic deformation region is fairly close to simple shear loading.

The samples for microstructural observation were cut parallel to the deformation direction by EDM, and metallographic specimens were prepared by standard mechanical polishing and etched in Kroll’s reagent. Scanning electron microscopy (SEM) and transmission electron microscopy (TEM) specimens were prepared from different loading conditions to allow a comparative characterization of the microstructure. The observation was focused on the center of the shear region (dotted circle shown in Figure 1). The samples for TEM observation were first polished to a thickness of about 50 μm, and then a 3-mm-diameter foil was carefully perforated from the samples, followed by ion-beam milling in a Gatan 691 (Gatan Inc., Las Positas Blvd., Pleasanton, CA, USA) precision ion-polishing system at 5 KeV with a final polishing step at 3 KeV of ion energy. Optical microscopy (OM) and SEM examinations were performed on LEICA DMI 3000M Kirana-05M (Leica Microsystems CMS GmbH, Wetzlar, Germany) and HITACHI S-4800 (Hitachi High-Technologies Corporation, Chiyoda-ku, Tokyo, Japan), respectively. TEM observations were conducted in a FEI Tecnai G^2^-F30 (FEI Corporation, Hillsboro, OR, USA) transmission electron microscope operating at 200 kV.

## 3. Results and Discussion

### 3.1. Mechanical Tests

When one-dimensional stress waves in the bars are achieved and the specimen is in a state of uniform stress, the histories of applied force *F* (distributing on the upper surface of the HS specimen) and punching depth *P_d_* in the specimen can be determined by:

(1) $$\begin{array}{ll} F_{s}\left(t\right) & =A_{0}E_{0}[\varepsilon_{i}(t)+\varepsilon_{r}(t)+\varepsilon_{t}(t)]/2\\ & =A_{0}E_{0}\varepsilon_{t}(t) \end{array},$$

(2) $$P_{d}\left(t\right)=-2C_{0}\int_{0}^{t}\varepsilon_{r}\left(t\right)dt,$$

where *A*_0_ is the cross-sectional area of the bars, and *E*_0_ and *C*_0_ are Young’s modulus and elastic bar wave speed of the bar material, respectively. Here, *ε_i_*(*t*), *ε_r_*(*t*) and *ε_t_*(*t*) represent incident, reflected and transmitted strain histories in the bars at the specimen ends, respectively.

Then, the shear stress (*τ_s_*), global shear strain (*γ*), local shear strain (*γ_loc_*) and local shear strain rate (${\dot{\gamma }}_{loc}$) of the HS specimen can be estimated as:

(3) $$\tau_{s}\left(t\right)=2\sqrt{5}E_{0}A_{0}\varepsilon_{t}\left(t\right)/\left(5A_{s}\right),$$

(4) $$\gamma =P_{d}/t_{D},$$

(5) $$\gamma_{loc.}=P_{d}/\left(3t_{M}\right),$$

(6) $${\dot{\gamma }}_{loc.}=v/({2t}_{M}),$$

where *A_s_* is the initial cross-sectional area of the specimen, and *t_D_* and *t_M_* are the designed width of the shear region (approximately 1 mm, dashed circle shown in Figure 1) and measured shear band width, respectively.

The shear stress can be converted into normal stress (*σ*) as:

(7) σ=2τ.

**Figure 2:**
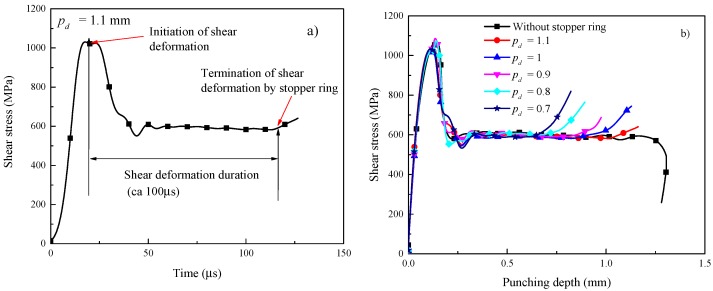
(**a**) Shear stress vs time for the HS sample deformed at a punching depth of 1.1 mm. A time window of approximately 100 µs indicating the period during shear band deformation; (**b**) shear stress vs different prescribed punching depths. Notice the upturn of the curves when prescribed displacement is reached.

The duration of shear deformation can be determined directly from the experimental data of the SHPB test, as shown in Figure 2a. The shear deformation duration is approximately 100 µs for the specimen deformed at a punching depth of 1.1 mm. The shear stress versus punching depth curves at all punching depths are shown in Figure 2b, where all of the curves exhibit oscillations due to reflection of waves at the specimen surfaces and stopper rings. In addition, these curves show a plateau for different punching depths, and a sharp increase in shear stress when the steel stopper ring is contacted. It is interesting to note that the flow stresses are almost a constant for different punching depths and are equal to approximately 600 MPa, indicating that the flow stress of Ti-55511 alloy is independent of the punching depth. Therefore, the corresponding normal stress of Ti-55511 alloy can be taken as 1200 MPa. However, in our previous work, the normal stress of Ti-55511 alloy is approximately 1500 MPa for cylindrical specimens under dynamic compression [23]. Hence, the corresponding normal stress in HS specimens is about 300 MPa lower than that in the cylindrical specimen. The observed discrepancy may be attributed to the geometrical imperfection of HS specimens.

### 3.2. Microstructural Characterization

**Figure 3:**
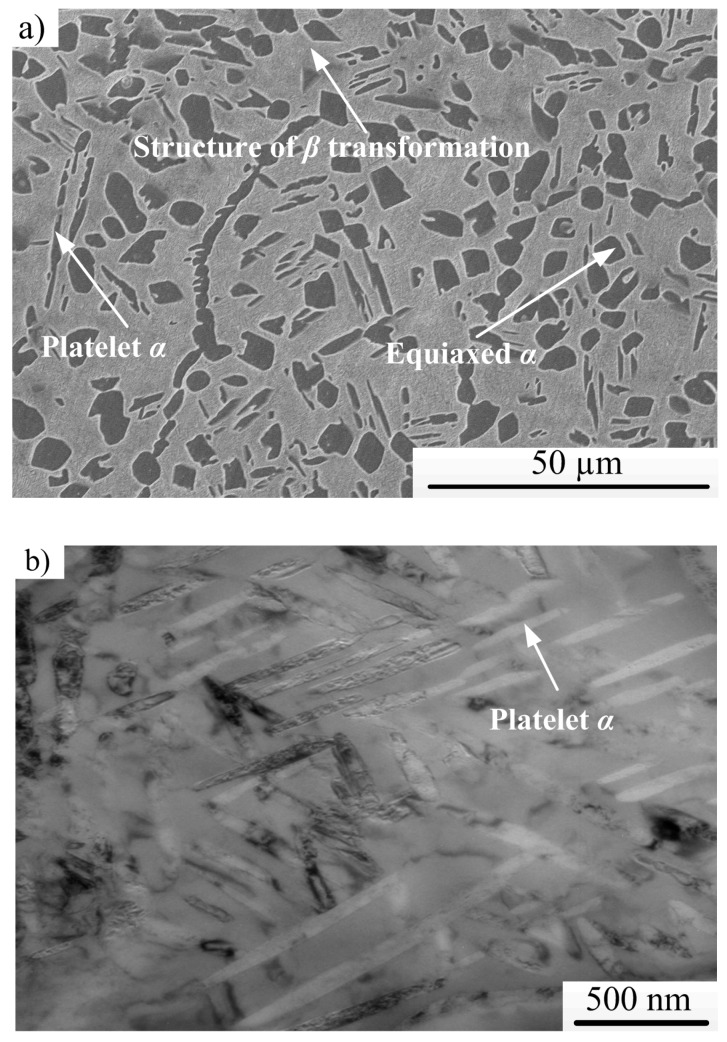
Initial microstructure of Ti-55511 alloy; (**a**) SEM micrograph and (**b**) TEM micrograph of structure of *β*-transformation.

A typical SEM micrograph of an undeformed specimen is depicted in Figure 3a. A higher-magnification TEM micrograph of the structure of *β*-transformation (matrix) is shown in Figure 3b, in which many thinner platelet α-phases can be observed. As shown in Figure 3, the initial microstructure of Ti-55511 alloy consists of the structure of *β*-transformation (matrix) and α-phase (platelet α and equiaxed α), and the size of equaxied α is approximately 4 μm.

**Figure 4:**
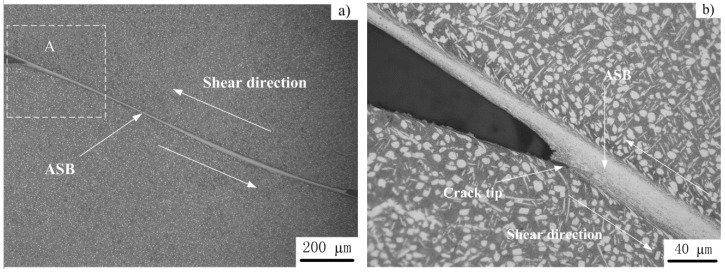

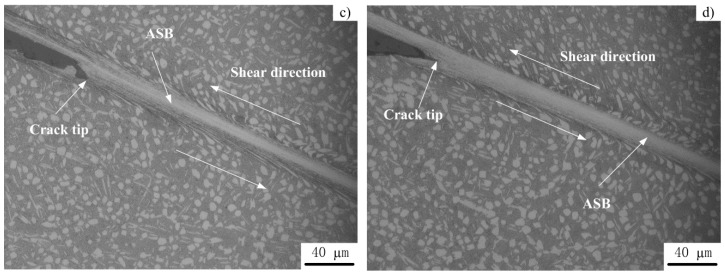
Typical optical micrographs of shear-deformation regions in HS specimens deformed at different punching depths (**a**) without stopper ring; (**b**) *P_d_* = 1.0 mm; (**c**) *P_d_* = 0.9 mm; (**d**) *P_d_* = 0.7 mm.

**Figure 5:**
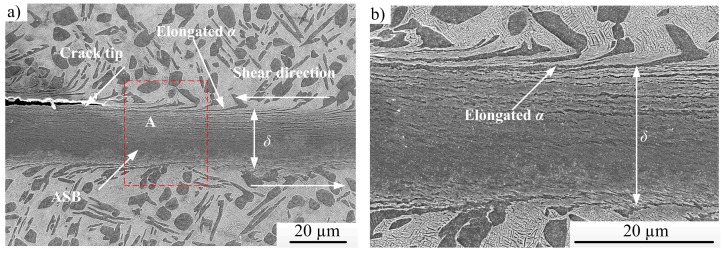
Typical SEM micrographs of shear-deformation regions in HS specimens deformed at *P_d_* = 0.8 mm: (**a**) a shear band and an associated crack and (**b**) higher magnification of A in Figure 5a.

Typical low-magnification optical micrographs of the well-developed localized shear bands are shown in Figure 4. Figure 4b–d are the higher magnifications of region “A” shown in Figure 4a. As shown in Figure 4, the values of shear band width range from 20–28 μm. Typical SEM micrographs of the shear-deformation region in the HS specimen deformed at a punching depth of 0.8 mm are show in Figure 5. Figure 5a shows an ASB with an associated crack, and a higher-magnification micrograph of region “A” in Figure 5a is shown in Figure 5b.

As shown in Figure 4 and Figure 5, α-phases adjacent to the ASB are elongated along the shear direction due to the strong shear deformation. The size of elongated “equaxied” α-phases along the shear direction is about 5–6 μm, larger than the initial size of 4 μm for the undeformed specimen (Figure 2a). With further deformation, the elongated α-phases break up into small structures within or close to the shear band. Moreover, a very distinctive boundary separates the shear band from the surrounding deformed structures. It should be noted that although the cracks extend into the shear band to some extent (Figure 4d), almost all cracks occurred at the shear band/matrix interface. Similar phenomena have been observed in Ti-55511 alloy [16,28,29].

**Figure 6:**
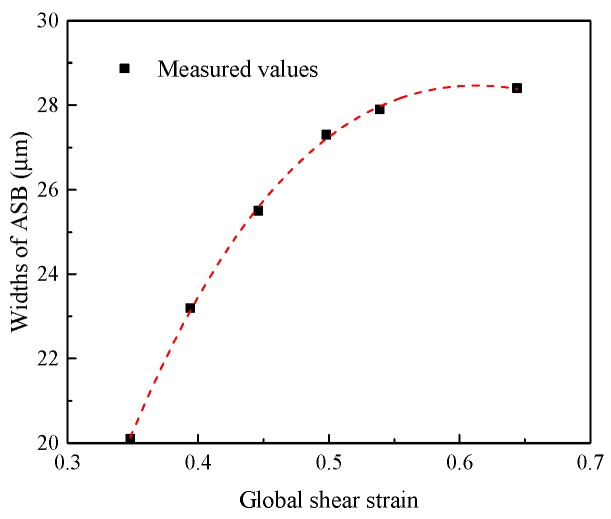
Widths of shear bands as a function of global shear strain.

The width of ASB for each loading condition is measured and plotted in Figure 6. It is shown that the width of ASB increases with the increase of global shear strain (punching depth), and tends to saturate at approximately 30 μm. The measured widths are compared with the values predicted using the equation proposed by Bai et al. [30] and Dodd and Bai [13], which will be discussed in Section 3.3.

**Figure 7:**
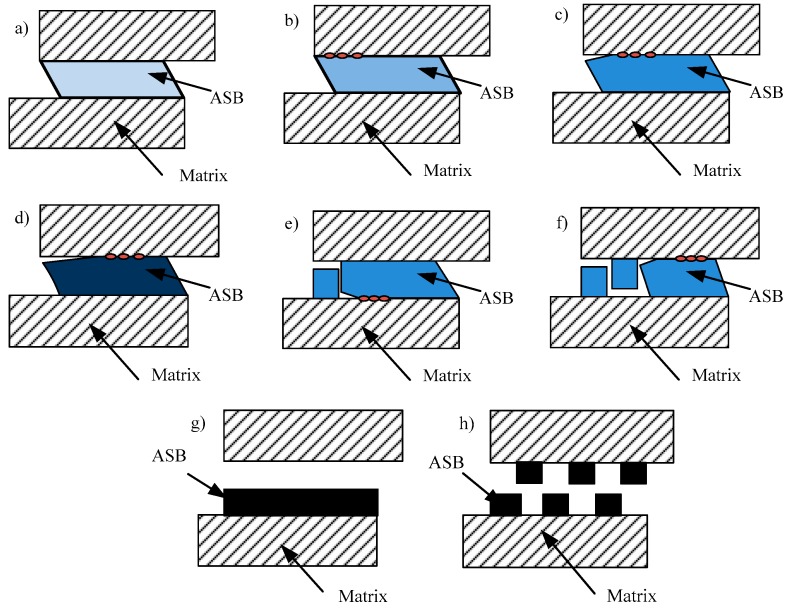
Schematic representation of the sequence of the microstructural evolution within an ASB for Ti-55511 alloy. (**a**): ASB formation; (**b**): microcracks formation (red points at the shear band/matrix interface); (**c**): adjoining microcracks coalesce; (**d**): crack propagation along the shear band/matrix interface; (**e**,**f**): crack propagated into the ASB; (**g**,**h**): ultimate fracture.

Combined with our previous work [16,29] and the microstructures shown in Figure 4 and Figure 5, the sequence of the microstructural evolution within an ASB for Ti-55511 alloy can be summarized, and Figure 7 is the schematic representation. As shown in Figure 7a, an ASB forms due to the occurrence of severe strain concentration in the shear region, and the grains or phases near the ASB are rotated and elongated along the shear direction (shown in Figure 4 and Figure 5). Then, microcracks are nucleated at the shear band/matrix interface (Figure 7b), and adjoining microcracks coalesce to a bigger crack (Figure 7c). With further deformation, the crack propagates along two ways. One is that the crack propagates along the shear band/matrix interface (Figure 7d) up to failure or fracture (Figure 7g). The other one is that the crack extends into the ASB and propagates along the shear band/matrix interface (Figure 7e,f) up to failure or fracture (Figure 7h). It is interesting to note that the features of ASB evolution in Ti-55511 alloy are similar to those of adhesive fracture. Hence, “adhesive fracture” can be identified as the dynamic failure mechanism for Ti-55511 alloy [29].

**Figure 8:**
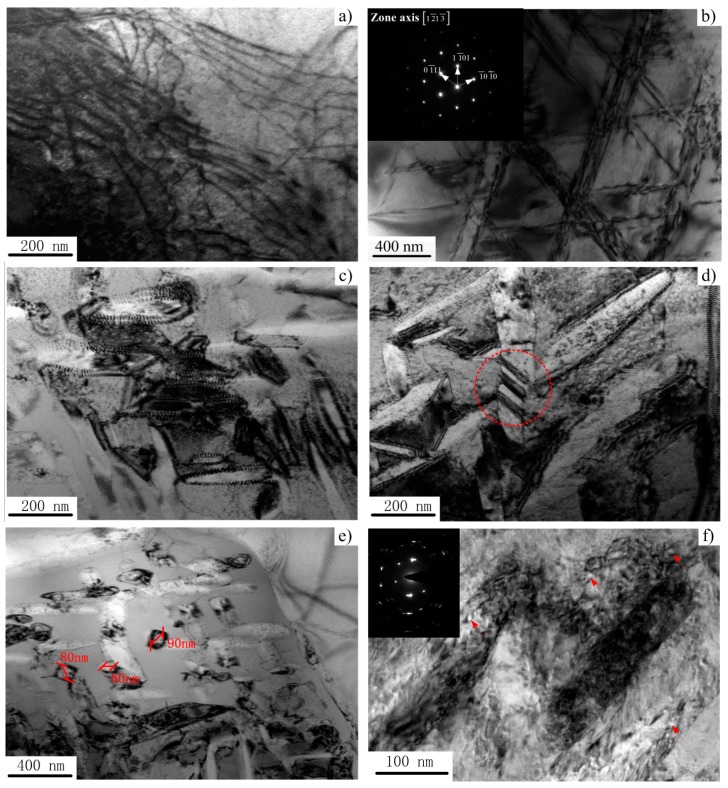
TEM micrographs of shear-deformation regions in HS specimens deformed at different punching depths: (**a**) *P_d_* = 0.7 mm, (**b**) *P_d_* = 0.8 mm, (**c**) *P_d_* = 0.9 mm, (**d**) *P_d_* = 0.9 mm, (**e**) *P_d_* = 1.0 mm and (**f**) without stopper ring.

The microstructure within and/or close to ASB was characterized by TEM, and characteristic microstructures of the shear region at different punching depths are shown in Figure 8. As shown in Figure 2b, the initial microstructure of undeformed Ti-55511 alloy shows low dislocation density in the crystal. Compared with the initial microstructure of Ti-55511 alloy, as shear deformation proceeds, planar parallel dislocations and dislocation dipoles can be observed (see Figure 8a). Due to the motion of dislocations along the shear direction, bamboo incidental dislocation boundaries form when the punching depth is increased to 0.8 mm (see Figure 8b). When the punching depth is further increased to 0.9 mm, cell structures form as the dislocations from various slip systems begin to tangle and pile up (see Figure 8c,d). These small areas or cells, outlined by broad boundaries, exhibit very few or no individual dislocations. Moreover, a dislocation pile-up group and stacking fault can be observed easily. The break-up of elongated cells can also be observed in Figure 8d (marked by the dotted circle). The tridimensional shape of the elongated cells is expected to be a “pancake”, since the observation was made on a plane that was parallel to the shear direction. A similar result has been reported by Meyers et al. [7]. With further deformation (*P_d_* = 1.0 mm), some grains and/or phases are broken up into subgrains with a size of approximately 60–90 nm. Apparently, these subgrains are essentially free of dislocations. For one shot test (sample forced sheared by split Hopkinson pressure bar without stopper ring), due to the severe shear deformation, the adjoining subgrains with a small misorientation between them will attempt to lower their surface energy by rotating into coincidence, thereby eliminating the low-angle boundary that separates them [27,31]. Due to the rotating and coalescence of adjoining subgrains, the nanograins with a size of about 6 nm can be observed (see Figure 8f). The selected area-diffraction pattern shows incomplete rings, which indicate the presence of dynamic recrystallization (DRX, marked by arrows). This is in accordance with the result reported by Rittel et al. [9].

Relying on the aforementioned analysis, the difference of the microstructures within the ASB of Ti-55511 alloy is dependent on the punching depth/plastic deformation. The sequence of plastic deformations taken place in the shear region can be summarized as: (a) occurrence of parallel planar dislocations; (b) formation of bamboo incidental dislocation boundaries; (c) formation of cells and/or elongated cells; (d) formation of subgrains; and (e) formation of nanograins.

Based on the criterion proposed by Derby [32] and Takeuchi and Argon [33], the recrystallized grain size can be estimated as:

(8) λ = KbG/σ,

with 1 < K < 15. G, *σ* and b are the shear modulus, applied stress and Burgers vector, respectively. For Ti-55511 alloy, G and b are 40 GPa and 2.3 × 10^−10^ m, respectively. *σ* is about 1.2 GPa. Thus, the estimated DRX size of Ti-55511 alloy ranges from 6–90 nm, which agrees well with our experimental observations.

Based on the adiabatic assumption, a large portion of the plastic work within a shear band is converted into heat to raise the local temperature [34]. The maximum temperature during the forced shear tests can be estimated as:

(9) $$T=\Delta T+T_{0}=\frac{\beta \int \tau d\gamma }{\rho C}+T_{0},$$

where *ρ* is the mass density, *C* is the specific heat, *T*_0_ is the ambient temperature and *β* is the fraction of plastic work converted to heat, which is taken as 0.9. For Ti-55511 alloy, *ρ* and *C* are 4625 kg/m^3^ and 523 J/(kg K), respectively. Here, *T*_0_ = 293 K.

The estimated maximum temperature in our tests is approximately 573 K. Such a temperature is much lower than those of *α*→*β* phase transformation (approximately 1163 K) and dynamic recrystallization (0.4 T_m_ [25], approximately 760 K). Hence, this observation indicates that thermal softening has a very minor effect on the onset of ASB and subgrain/nanograin formation. Similar findings have also been reported by Rittel et al. [9] and Clos et al. [35]. Therefore, the nucleation mechanism for DRX can be assumed as strain-induced boundary migration and subgrain rotation and coalescence. Similar phenomena have been reported in copper [25,27] and TA2 [7].

### 3.3. Estimation of ASB Width

The width of ASB has been satisfactorily predicted with perturbation analyses [13,14]. The contribution of heat conduction to the thickness of a shear band was included in these analyses [30]. A very simple estimation of the ASB width has been given by Bai et al. [30] and Dodd and Bai [2,13,14],

(10) $$\delta =2\sqrt{\frac{\lambda T_{\max}}{\beta \tau {\dot{\gamma }}_{loc}}},$$

where *δ* is the shear band width, and *λ* and *T*_max_ are the thermal conductivity and maximum temperature within the shear band, respectively. For Ti-55511 alloy, *λ* is equal to 9.21 W/(m K).

**Figure 9:**
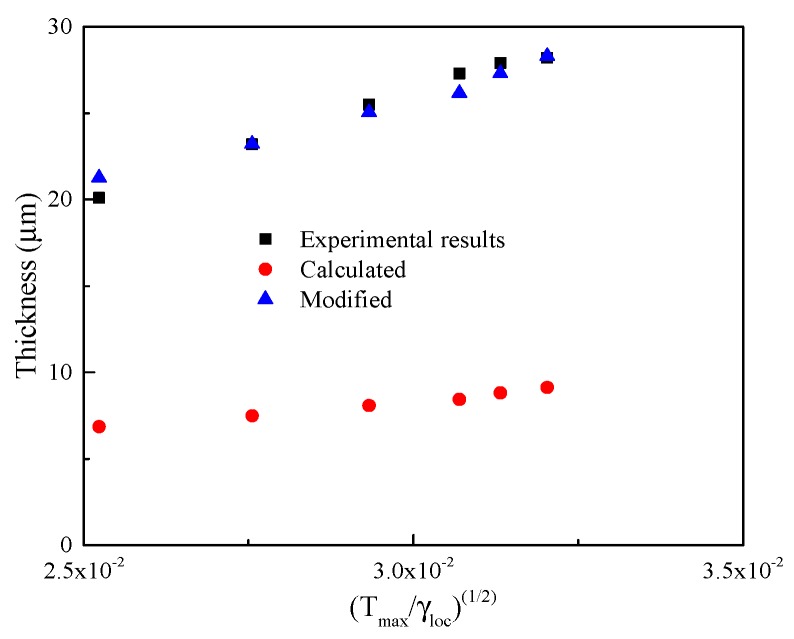
Width of ASB in Ti-55511 alloy (HS specimens) as a function of the maximum temperature and local shear strain rate. The theoretical predictions using the original Formula (10) and the modified one (11) are given for comparison.

The applied shear stress of Ti-55511 alloy in HS specimens is estimated as a constant of 600 MPa. Then, the width of ASB can be considered as a linear function of $\sqrt{T_{\max}/\gamma_{loc}}$. The calculated width of ASB and the ratio of the calculated and measured widths of ASB for each test are listed in Table 2. Figure 9 shows the width of ASB plotted against $\sqrt{T_{\max}/\gamma_{loc}}$. It is shown that the widths of ASB predicted using Equation (9) are much smaller than the experimental results.

It should be pointed out that the width of ASB predicted using Equation (9) agrees well with the experimental result in our previous work, in which the measured ASB width for Ti-55511 alloy is about 6–9 μm in compression tests of cylindrical specimens [23]. Meyers et al. [7] and Chen et al. [15] calculated the ASB width for different materials in HS specimens, and gave different results. A three-fold difference between experimentally observed and calculated results was observed in TA2 by Meyers et al., while Chen et al. reported that the experimentally observed width was slightly higher than that calculated for tantalum. In the present study, the measured widths of ASB are about two-times larger than the calculated results. Though Wang et al. [28] did not calculate the thickness of the ASB using Equation (9), in their work, the calculated width of ASB was approximately 1/3 of the measured one (H4 sample). Hence, for Ti-55511 alloy, the measured thickness of ASB has about a three-fold difference from the calculated results. Therefore, the calculation of ASB width for HS specimens can be modified as:

(11) $$\delta_{M}=2k\sqrt{\frac{\lambda T_{\max}}{\tau \gamma_{loc}}},$$

where *k* is the coefficient.

Relying on the aforementioned analysis, the values of k for TA2, tantalum and Ti-55511 alloy are 1/3, 1 and 3, respectively. The discrepancy may be attributed to the material itself. Further investigation is needed to clarify this issue. It should be noted that the result obtained in the present study cannot be generalized to other metallic materials until further tests are carried out.

## 4. Conclusions

A series of dynamic forced shear tests were carried out on Ti-55511 alloy at 293 K by means of the SHPB technique using HS specimens combined with the “strain-frozen” technique, and the microstructure of the localized shear region was examined. According to the experimental findings, the following conclusions can be drawn:

(a) The flow stress in the HS specimens of Ti-55511 alloy remains a constant of about 600 MPa, and is independent of punching depth.
(b) The width of the shear band increases with the increase of punching depth and tends to saturate at 30 μm, and the estimation of the ASB width in HS specimens has been modified.
(c) Thermal softening has a minor effect on the onset of ASB and DRX formation, and the nucleation mechanism for DRX is strain-induced boundary migration and subgrain rotation and coalescence.
(d) For Ti-55511 alloy, the features of microstructural evolution in high-strain-rate loading situations are similar to those of adhesive fracture, and the concept of adhesive fracture is proposed as the dynamic failure mechanism for Ti-55511 alloy.

## Figures and Tables

**Table 1:** Chemical composition of Ti-5Al-5Mo-5V-1Cr-1Fe alloy (wt %).

Al	Mo	V	Cr	Fe	C	N	H	O	Zr	Si	Ti
5.50	4.82	4.82	1.02	1.02	0.02	0.03	0.001	0.1	0.15	0.10	balance

**Table 2:** Summary of the global shear strain, local shear strain rate, maximum temperature and ASB width.

*P_d_*/mm	*v*/ms^−1^	*t_M_/*μm	*t_D_*/mm	*γ*	*γ_loc_*	${\dot{\gamma }}_{\mathrm{loc}}$/s^−1^	*T*_max_/K	*δ/*μm	*δ/t_M_*
0.7	23.8	20.1	1.01	0.693	34.83	5.92 × 10^5^	454	6.86	2.93
0.8	23.7	23.2	1.03	0.777	34.48	5.11 × 10^5^	467	7.49	3.10
0.9	23.7	25.5	1.02	0.882	35.29	4.65 × 10^5^	494	8.08	3.16
1	23.7	27.3	1.01	0.990	36.63	4.34 × 10^5^	504	8.44	3.23
1.1	23.5	27.9	1.04	1.058	39.43	4.21 × 10^5^	532	8.81	3.17
Without stopper ring	23.8	28.2	1.02	1.196	46.10	4.22 × 10^5^	573	9.13	3.09
